# OliveTreeCrownsDb: A high-resolution UAV dataset for detection and segmentation in agricultural computer vision

**DOI:** 10.1016/j.dib.2025.111515

**Published:** 2025-03-22

**Authors:** Youness Hnida, Mohamed Adnane Mahraz, Ali Achebour, Ali Yahyaouy, Jamal Riffi, Hamid Tairi

**Affiliations:** a L3IA - Laboratory of Computer Science, Innovation, and Artificial Intelligence, Faculty of Sciences Dhar El Mahraz, Sidi Mohamed Ben Abdellah University, Fez, Morocco; b Geographic Information Technology and Space Management Team (ETIGGE), Laboratory of Communication, Education, Digital Uses, and Creativity, Faculty of Arts and Humanities, Mohamed 1st University, Oujda, Morocco; c Research and Development Department, Drone Globe, Rabat, Morocco; d LaMSN - La Maison des Sciences Numériques, USPN, Paris, France

**Keywords:** OliveTreeCrownsDb, Drone, Olive tree crowns, Precision agriculture, Computer vision

## Abstract

This article introduces OliveTreeCrownsDb, a comprehensive dataset of high-resolution images captured by a DJI Phantom 4 RTK drone. The dataset includes 46 images covering an entire olive farm, focusing on the detection and analysis of olive tree crowns and supporting segmentation tasks. Each image is accompanied by detailed metadata, such as focal distance, capture altitude, GPS coordinates, and other essential parameters for accurate tree mapping and localization. OliveTreeCrownsDb is publicly accessible, promoting research in precision agriculture, including tree crown detection, segmentation, geometric shape analysis, automation, yield estimation, and computer vision applications. It facilitates the development of innovative algorithms to optimize resource allocation and improve crop management. By enabling studies on tree crown analysis and farm monitoring, OliveTreeCrownsDb advances agricultural technologies and enhances management practices in olive cultivation.

Specifications Table

**Table:** 

Subject	Computer Vision Applications (CV)
Specific subject area	OliveTreeCrownsDb, Deep Learning, Object Detection and Segmentation, Agricultural Computer Vision
Type of data	RGB Images, LASer Format (LAS), Tag Image File Format (TIFF), Spatial Data (KML)
Data collection	The data was collected using a DJI Phantom 4 RTK drone equipped with the FC6310 camera, which provides a resolution of 5472 × 3648 pixels. The flight paths were carefully planned to ensure full coverage of the mining site in Meknas, including the distribution of objects and processing infrastructure. During this data collection, 46 georeferenced images were captured in approximately 3 min of flight, utilizing the drone's integrated GPS system. The study area is located at coordinates 33°53′17"N, 5°25′22"W, with a flight altitude of 74.7 meters. The ground sample distance (GSD) of the images is 1.78 cm/pixel, ensuring high precision in the analysis of geographical details.
Data source location	City / Town / Region: Meknas farm site Country: Morocco The GPS coordinates of the olive farm are 33°53′17"N 5°25′22"W, or in decimal format: 33.88802°N, -5.42281°W.
Data accessibility	Repository name: OliveTreeCrownsDb Data identification **number**: doi: 10.17632/xym8rd2srf.2 Direct URL to data: Instructions for accessing these data: https://data.mendeley.com/datasets/xym8rd2srf/2
Related research article	none

## 1 Value of the Data

The OliveTreeCrownsDb dataset is a valuable resource for research in computer vision and precision agriculture. Here are the key aspects that highlight its importance:

• Unique and Specialized Source: OliveTreeCrownsDb offers an exclusive high-resolution dataset specifically aimed at studying olive tree crowns. This dataset is rare and crucial for targeted analysis in this specific agricultural domain.
• Applications in Precision Agriculture: The dataset enables the development of models for detecting, evaluating, and optimizing tree crowns, promoting the estimation of olive tree health and productivity. This resource supports modern agricultural practices and efficient crop management.
• Georeferenced Data for Localization Research: With precise GPS information included in each image, OliveTreeCrownsDb is ideal for mapping and geolocation research. These metadata allow for detailed spatial analysis and are applicable in precision agriculture.
• Development of Computer Vision and AI Algorithms: OliveTreeCrownsDb is a key resource for training AI algorithms in object detection and classification within the agricultural field, advancing automation and computer vision.
• Accessibility and Contribution to the Community: This dataset is made publicly available, encouraging collaboration among researchers and fostering innovation in the field.OliveTreeCrownsDb provides open access to high-quality data, promoting transparency and knowledge sharing.

## 2 Background

The OliveTreeCrownsDb dataset was developed to address the need for high-quality visual data in computer vision, particularly in agricultural applications. Unlike many existing datasets that lack precision, OliveTreeCrownsDb provides georeferenced, drone-captured images with detailed information on olive tree crowns. This level of accuracy enables more effective crop analysis and real-time monitoring, supporting faster decision-making and the automation of agricultural tasks. As drone technology becomes more prevalent in agriculture, OliveTreeCrownsDb plays a key role in improving crop assessment and resource management, fostering advancements in computer vision while meeting the demands of sustainable and smart agricultural practices.

## 3 Data Description

**Fig. 1:**
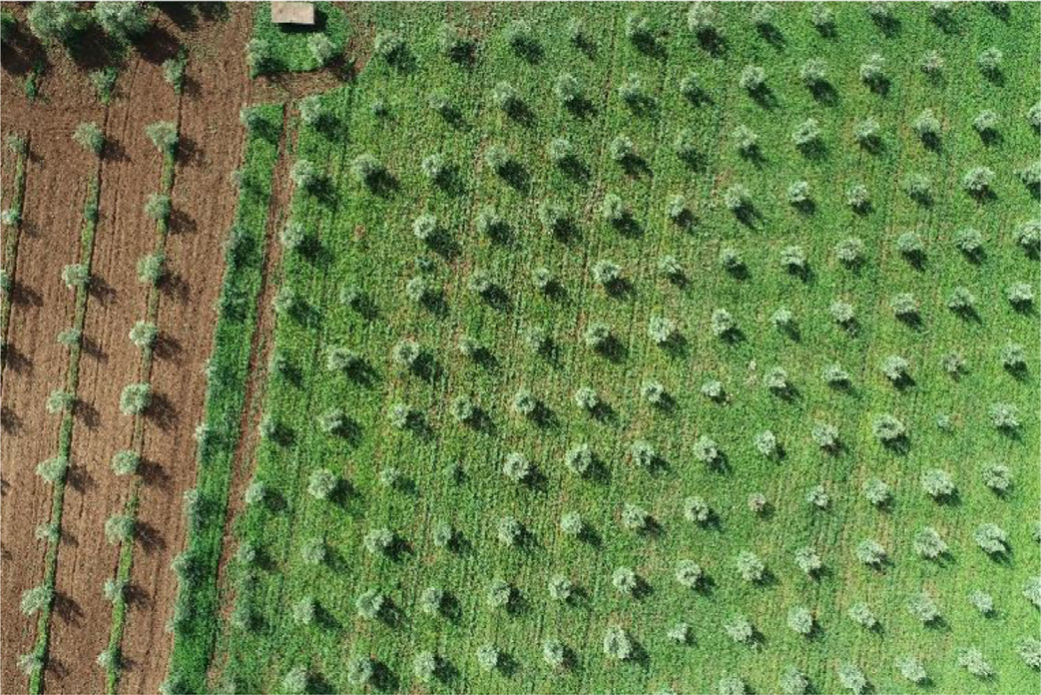
Example of high-resolution aerial image of the field.

In this article, we present the OliveTreeCrown dataset, collected using a DJI Phantom 4 RTK drone over a 4.22-hectare farm in the Meknes region of Morocco. The data collection was conducted at a flying altitude of 74.7 meters, capturing 46 RGB images with an average overlap of 85% and a ground resolution of 1.78 cm, allowing for detailed observation of objects. The olive trees on the farm are planted with a spacing of 6 × 6 meters, enabling analysis of their distribution and growth patterns. Notably, the farm features high-density planting along the borders, where traditional olive cultivations are densely arranged, and some areas contain wild olive trees with closer spacing.For multi-scale analysis, these images were segmented into grids of (3 × 3), (6 × 6), and (9 × 9), and objects were annotated. In addition to image annotations, the OliveTreeCrown dataset includes geospatial files generated via Agisoft Metashape: a LAS file and a DEM file. The LAS file provides an accurate 2D point cloud model of the terrain and objects, while the DEM file offers a detailed view of the terrain's elevation and relief. These files are essential for understanding the topography and enable in-depth analyses for precision agriculture and cartographic applications. Together, they complement the annotations by providing precise information on the 3D positioning and distribution of olive trees, allowing for an analysis of how slopes and terrain features influence tree distribution. Fig. 1 illustrates the images collected by the drone. Table 1 summarizes the key aspects of the dataset, including the number of high-resolution images captured, the standardized file naming format, and image resolution. Table 2 presents the technical specifications of the camera used for data collection, including details on the camera model, sensor type, and flying altitude. Table 3 offers a comparative analysis of the datasets used in aerial image processing.

**Table 1:** The key components of the dataset.

Folder	Filename	Feature	Description
OliveTreeCrownsDb	DJI_XXXX.JPG	Image files	46 high-resolution images
Other Data	DEM_Meknas.tif	TIF file	Digital Elevation Model
	Meknas_point_cloud.las	LAS file	3D point cloud file
	DEM_Contour.kml	Kml file	Base Contour Elevation
Images without Crowns	XXX.jpg	Image files	RGB images (0 – 760)
Annotation/1 × 1/Images	XXX.jpg	Image files	RGB images (0 – 46)
Annotation/3 × 3/Images	XXX.jpg	Image files	RGB images (0 – 388)
Annotation/6 × 6/Images	XXX.jpg	Image files	RGB images (0– 1471)
Annotation/9 × 9/Images	XXX.jpg	Image files	RGB images (0 – 3177)
Annotation/1 × 1/label_voc	XXX.xml	XML files	VOC format (0 – 46)
Annotation/1 × 1/label	XXX.txt	TXT files	YOLO format (0 – 46)
Annotation/3 × 3/label_voc	XXX.xml	XML files	VOC format (0 – 388)
Annotation/3 × 3/label	XXX.txt	TXT files	YOLO format (0 – 388)
Annotation/6 × 6/label_voc	XXX.xml	XML files	VOC format (0 – 1471)
Annotation/6 × 6/label	XXX.txt	TXT files	YOLO format (0 – 1471)
Annotation/9 × 9/label_voc	XXX.xml	XML files	VOC format (0 – 3177)
Annotation/9 × 9/label	XXX.txt	TXT files	YOLO format (0 – 3177)

**Table 2:** Parameters of the data acquisition mission.

Parameter	Drone Model	Camera type	Focal Length	Aperture	Flight Altitude	GSD	Flight Speed	Image Format
Description	DJI Phantom 4 RTK	FC6310R	8.8 mm	f/5	74.7 m	1.78 cm/pixel	5 m/s	JPG, TIF

**Table 3:** Comparison of datasets for aerial image analysis.

Dataset	Images	Size	Tasks	Image Size (Pixels)	Channels	Resolution (GSD)
OAM-TCD (Tree Crown) [1]	∼5072 images	3.55 GB	Segmentation of individual tree crowns	2048 × 2048	RGB	10 cm/pixel
25kTrees (Tree Crowns) [2]	∼25,000 images	1.09GB	Segmentation of individual tree crowns	-	RGB	20 cm/pixel
NeonTreeEvaluation Benchmark [3]	6,000 images	1,8GB	Segmentation and detection	8,984 × 6,732	RGB	1 cm/pixel
OliveTreeCrownsDb (our)	5842 images	2.11GB	crown tree detection and mapping	5472 × 3648	RGB	1.78 cm/pixel

## 4 Experimental Design, Materials and Methods

### 4.1 Flight planning and data acquisition

In our project, we carefully planned the flight mission to ensure optimal data collection. The process began with selecting a suitable farm based on specific criteria, such as the number of trees planted and their spacing, as these factors significantly influenced the quality of the data and the efficiency of the acquisition process. We also accounted for the tree species present, ensuring they were relevant to the objectives of our study. Additionally, we chose a day with favorable weather conditions to capture high-quality images. Conducting the flight in clear weather, free from strong winds or precipitation, minimized interference and ensured reliable data acquisition.

### 4.2 Georeferencing

Georeferencing was a vital component of our data acquisition process. We employed GPS technology to precisely map the drone's position during the flight, ensuring that each captured image was accurately linked to its geographic coordinates. This geospatial information significantly enhanced the utility of the images, enabling advanced analyses such as tree health monitoring and growth assessment over time. By integrating GPS data, we ensured our dataset was both visually detailed and spatially accurate, providing a robust foundation for comprehensive analysis and valuable insights.

### 4.3 Safety protocols

Safety protocols were a critical component of both the flight planning and data acquisition phases, particularly given the presence of overhead power lines on the studied farm. Before the flight, we conducted a thorough risk assessment to identify potential hazards, including obstacles such as power lines, as well as the presence of people or animals nearby. Special attention was given to planning the drone's trajectory to avoid any interference with the power lines. Additionally, drone operators were well-trained and equipped with emergency procedures tailored to this environment. During the flight, team members maintained constant communication and closely monitored the drone's path to ensure compliance with safety guidelines. By integrating these specific considerations into our protocols, we minimized risks and ensured the safety of both the team and the environment throughout the data acquisition process.

### 4.4 Data processing

**Fig. 2:**
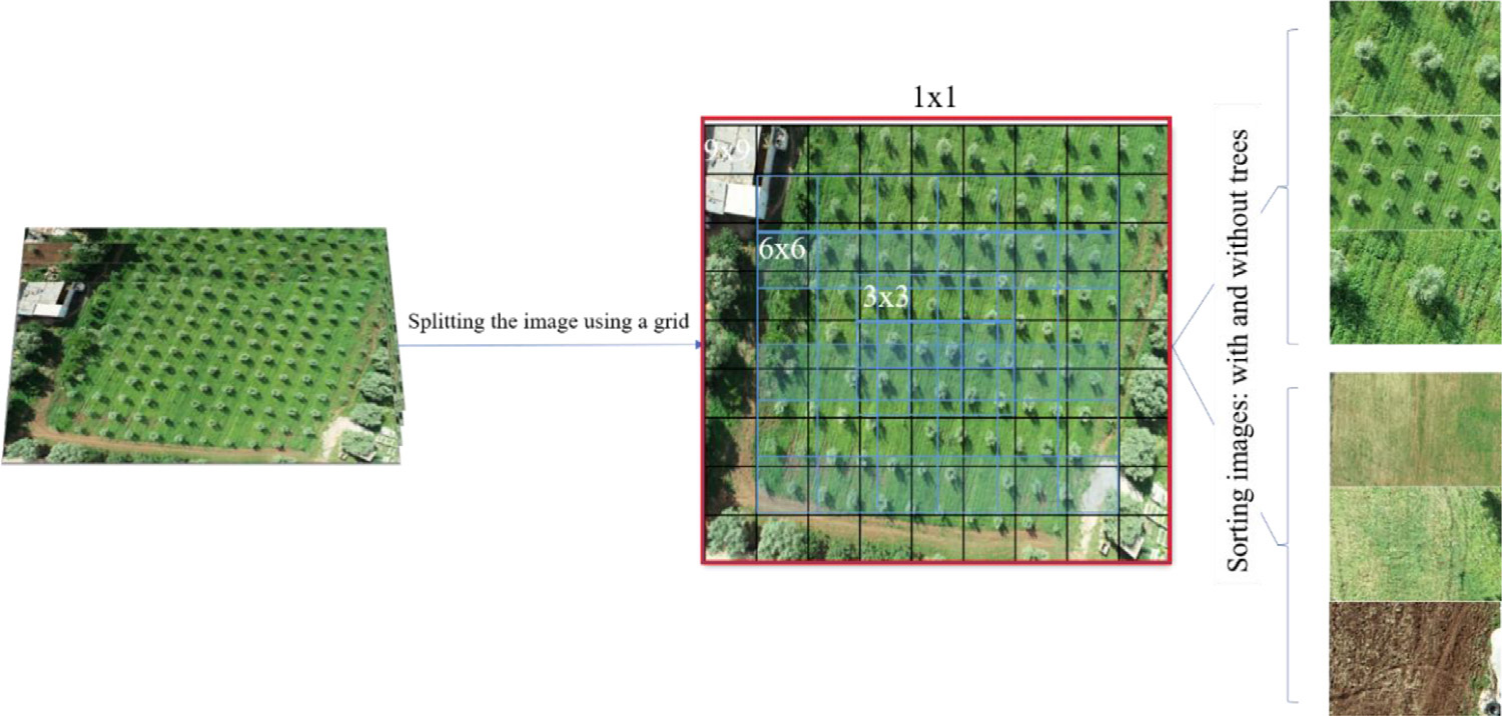
Grid segmentation and sorting for olive crown detection using UAV imagery.

For our study, we collected 48 high-resolution images, each measuring 5472 × 3648 pixels, covering the designated study area. To enable analysis at various levels of detail, we used the imgonline tool [4] to segment each original image (1 × 1) into smaller grids, including (3 × 3), (6 × 6), and (9 × 9) configurations. This segmentation approach allows us to explore the data at different scales, capturing a broad view with the (1 × 1) original image and increasingly detailed information with smaller grid sizes. This multi-scale segmentation method is especially beneficial for models that rely on layered analysis, as it provides deeper insights by revealing features across different levels of detail. By examining the images in multiple segment sizes, we can enhance the model's accuracy in detecting and distinguishing tree crowns across varying scales. Fig. 2 illustrates the proposed methodology. Beginning with the original image (1×1), grid-based segmentation is applied to generate (3×3), (6×6), and (9×9) tiles. These segments are then categorized based on the presence or absence of olive tree crowns, allowing for efficient dataset structuring in detection tasks. This multi-scale segmentation strategy enhances olive crown identification in UAV imagery by enabling spatial analysis across various resolutions.

## 5 Image Processing

### 5.1 Data annotation

**Fig. 3:**
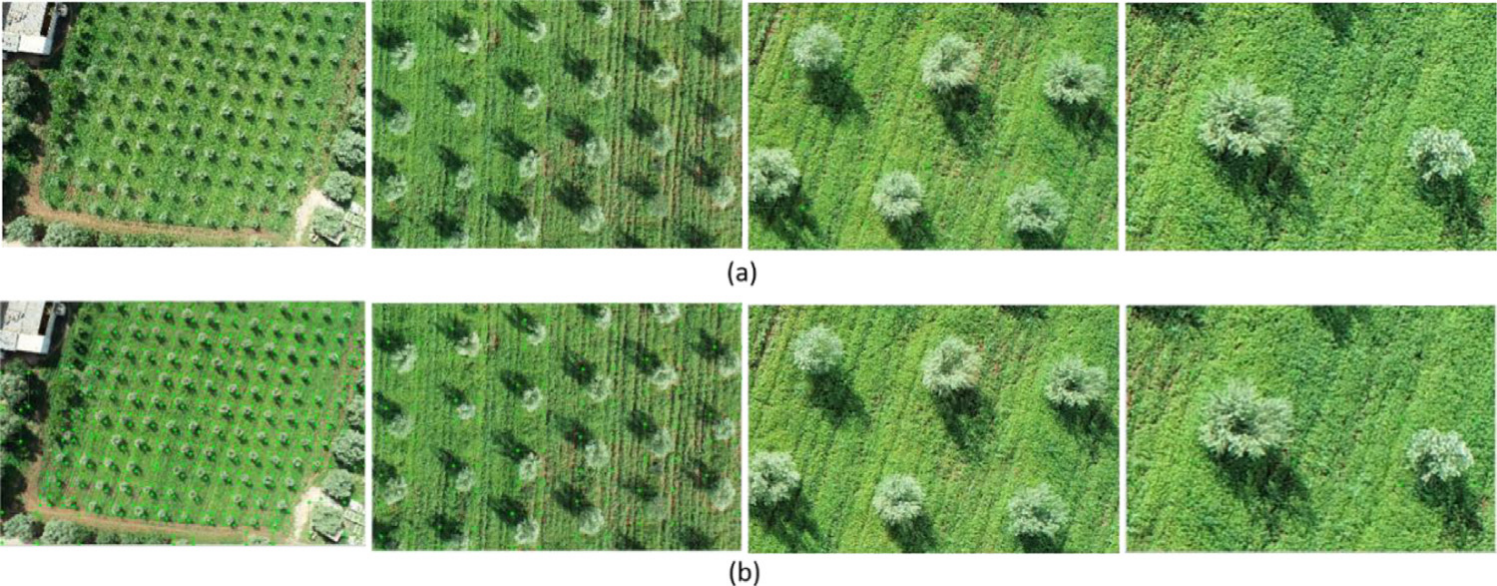
Different grid sizes (1 × 1, 3 × 3, 6 × 6, and 9 × 9) Visualization: a) Field image, b) Image annotation.

In this study, we analyzed 48 high-resolution images (5472 × 3648 pixels) of the study area. To address memory limitations, each image was divided into smaller segments using grid configurations of (9 × 9), (6 × 6), and (3 × 3), resulting in varying segment sizes. Manual annotation was performed with the LabelImg tool [4], which generated text files named to match their corresponding images. Each file contains details of annotated objects in the format (x, y, w, h), where x and y represent the coordinates of the bounding box center, and w and h indicate its width and height. Each line in the file represents a unique object in the image. This annotation process identified a total of 33,448 tree crowns across all segments. Special attention was given to trees located at the edges of segments, as including these edge trees is essential to ensure the model learns to detect trees even when they appear partially a common scenario in real-world applications where trees are not always isolated or fully visible within each image as shown in Fig. 3. Considering edge trees enhances the model's robustness, allowing it to recognize tree crowns at segment boundaries and thereby providing better spatial coverage. This approach enriches the training data, making the model more effective in complex scenarios, particularly in dense or mosaic-like environments where trees may only be partially visible in the imagery.

We present the image splitting analysis in Table 4, which outlines the number of images, grid configurations, segment dimensions, and annotation distribution. The parameters are defined as follows: Img represents the total number of original images used for each image splitting type; Type refers to the splitting type, where (1 × 1) corresponds to the original images without splitting, and (3 × 3), (6 × 6), and (9 × 9) represent splits into 9, 36, and 81 segments per image, respectively. Grid indicates the number of segments generated per image for each type, while Seg shows the total number of segments across all images after splitting. Dim specifies the dimensions of each segment. Empty refers to the segments without any annotated objects, and Annotated lists segments containing at least one annotated object. Finally, Annotations represents the total number of annotated objects for each splitting type.

**Table 4:** Analysis of image splitting: types, grids, dimensions, and annotation distribution.

Img	Type	Grid	split	Dim	Empty	Annotated	Annotation
46	1 × 1	1	46	5472 × 3648	0	46	6367
46	3 × 3	9	414	1824 × 1216	26	388	7316
46	6 × 6	36	1656	912 × 608	185	1471	9049
46	9 × 9	81	3726	608 × 405	549	3177	10716

### 5.2 Point cloud and Digital Elevation Model production

We used Agisoft Metashape [4] to create a Digital Elevation Model (DEM) and a digital orthophoto map mosaic from aerial images [[5], [6], [7]]. First, we imported 46 high-resolution images from drone flights into the software. The images were aligned by matching common points between them. This step was important for building an accurate model. After that, a dense point cloud was created, which provided detailed information about the terrain.

**Fig. 4:**
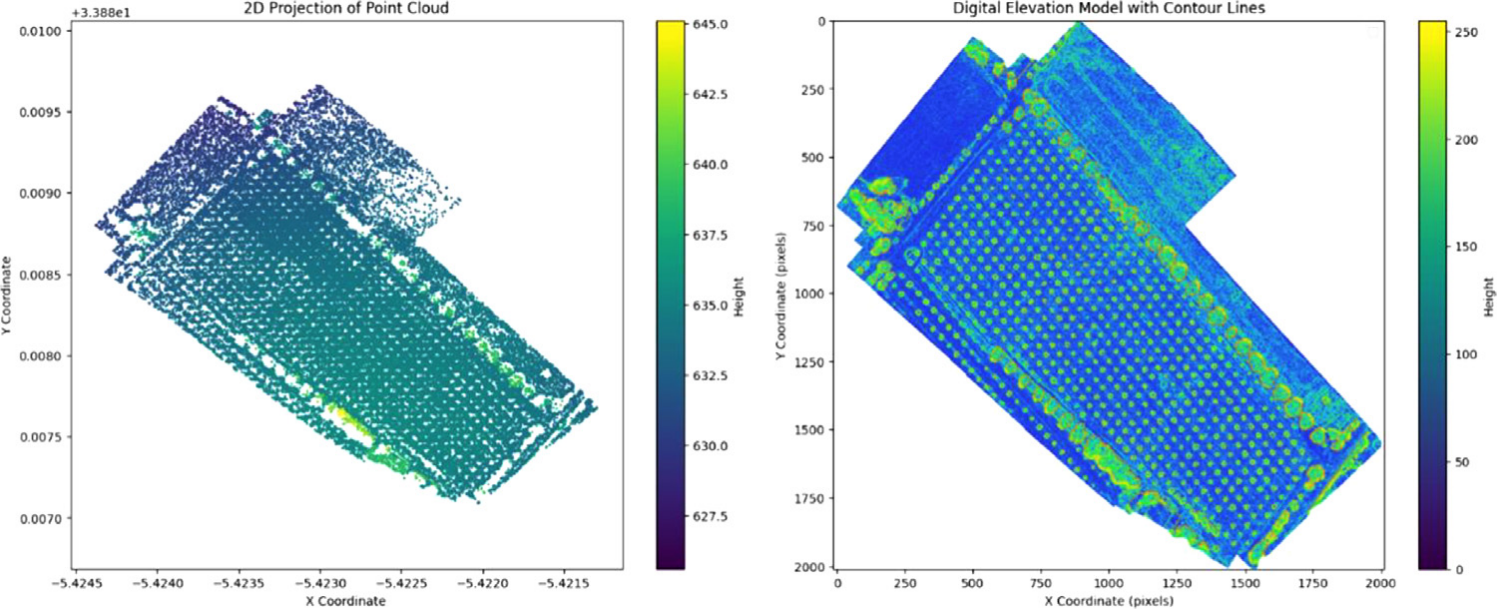
2D projection of point cloud and Digital Elevation Model with contour lines for terrain analysis.

The DEM, shown on the right in Fig. 4, represents the bare-earth surface by excluding objects like trees and buildings, thereby highlighting the topography [8]. The left side of Fig. 4 displays a 2D projection of the dense point cloud, which provides valuable depth information for the terrain's elevation, making the DEM more precise. Additionally, contour lines were applied to the DEM, further enhancing the interpretation of elevation variations across the area.

This combination of point cloud and DEM, with contouring, offers a comprehensive understanding of the landscape, supporting better site analysis and decision-making. The texture-mapped mosaic generated from the original images adds clarity and detail to the final model [9].

## Limitations

Not applicable.

## Ethics Statement

The authors confirm adherence to ethical standards throughout this research. No human, animal, or social media data were involved. All data were obtained with proper consent and in compliance with ethical guidelines. The study has no conflicts of interest and was conducted following institutional and regulatory ethical principles.

## CRediT authorship contribution statement

**Youness Hnida:** Conceptualization, Methodology, Software, Data curation, Writing – review & editing, Writing – original draft. **Mohamed Adnane Mahraz:** Supervision, Project administration, Investigation, Validation, Writing – review & editing. **Ali Achebour:** Software, Resources. **Ali Yahyaouy:** Formal analysis, Writing – review & editing. **Jamal Riffi:** Investigation, Visualization. **Hamid Tairi:** Supervision.

## Data Availability

Mendeley DataOliveTreeCrownsDb: A High-Resolution UAV Dataset for Detection and Segmentation in Agricultural Computer Vision (Original data).
